# Ultrasound-Guided Femoral Vascular Access for Percutaneous Coronary and Structural Interventions

**DOI:** 10.3390/diagnostics13122028

**Published:** 2023-06-11

**Authors:** Iosif Xenogiannis, Charalampos Varlamos, Thomas R. Keeble, Andreas S. Kalogeropoulos, Grigoris V. Karamasis

**Affiliations:** 1 Cardiology Department, Attikon University Hospital, Medical School, National and Kapodistrian University of Athens, 124 62 Athens, Greece; 2 Department of Cardiology, Mitera General Hospital, 151 23 Athens, Greece; 3 Department of Cardiology, Essex Cardiothoracic Centre, Basildon SS16 5NL, UK

**Keywords:** ultrasound-guided access, femoral artery, coronary angiography, percutaneous coronary interventions (PCI), transaortic valve implantation (TAVI)

## Abstract

Radial access has largely substituted femoral access for coronary interventions. Nevertheless, the femoral artery remains indispensable for gaining access to structural and complex percutaneous coronary interventions such as transcatheter aortic valve implantation and chronic total occlusion interventions, respectively. Ultrasound-guided femoral puncture is a broadly available, inexpensive, and relatively easy-to-learn technique. According to the existing evidence, ultrasound guidance for gaining femoral access has improved the effectiveness and safety of the technique. In the present paper, we sought to review the current literature in order to provide the reader with up-to-date data regarding the benefits of ultrasound-guided femoral access compared with the conventional technique as well as describing the state-of-the-art technique for gaining femoral access under ultrasound guidance.

## 1. Introduction

For years the femoral artery was the preferred site of access for percutaneous coronary interventions. However, several randomized controlled trials (RCTs) showed a clear advantage of radial over femoral artery access in terms of bleeding and, most importantly, survival leading the contemporary guidelines to support the usage of the radial artery for percutaneous coronary interventions (PCI) as the by default technique [1,2,3,4]. On the other hand, there are cases where access to the coronary arteries through the radial artery can be either very cumbersome (i.e., extreme subclavian artery tortuosity, presence of arteria lusoria for the right arm) or impossible (i.e., totally occluded arteries of the upper extremities) leaving femoral access as the only solution. In addition, the femoral artery can provide better support that is of paramount importance, especially for the most challenging lesions such as complex chronic total occlusions. In the field of structural heart interventions, the femoral artery is the preferred access site for procedures such as transcatheter aortic valve implantation (TAVI) [5,6]. Although older interventionalists were performing practically all their procedures via the femoral artery, the newer generation of interventional cardiologists is much more familiar with the use of the radial artery performing the majority of their procedures through it, being less experienced with the puncture of femoral artery compared with the older generation [7]. Nevertheless, expertise in femoral artery access is necessary to be maintained among interventional cardiologists, given the fact that it is the preferred or the only site of access for a variety of clinical scenarios, as described earlier. Since femoral access is associated with a higher percentage of access complications in comparison with radial access, every possible effort should be made to optimize the outcomes of this procedure. Ultrasound used to guide femoral artery puncture has significantly aided in this direction since it has been shown to be safer compared with anatomical landmarks-guided or fluoroscopy-guided femoral access. In this review, we sought to provide the available data that support the use of ultrasound for femoral access and describe the technique in a detailed but simple manner.

## 2. Existing Evidence Supporting the Use of Ultrasound-Guided Femoral Access

### 2.1. Ultrasound-Guided Femoral Access for PCI

Multiple studies have evaluated the potential advantage of ultrasound usage in gaining femoral access versus the conventional technique. The prospective, multicenter RCT FAUST (Femoral Arterial Access with Ultrasound Trial) was a landmark study on the field, randomizing 1004 patients to fluoroscopic or ultrasound guidance [8]. Ultrasound-guided access resulted in an improved first-pass success rate (83% vs. 46%, *p* < 0.0001), reduced number of attempts (1.3 vs. 3.0, *p* < 0.0001), reduced risk of venipuncture (2.4% vs. 15.8%, *p* < 0.0001), and reduced median time to access (136 s vs. 148 s, *p* = 0.003). In addition, patients in the ultrasound guidance group experienced a reduced number of vascular complications (1.4% vs. 3.4% *p* = 0.04). Finally, ultrasound increased access success in cases of common femoral bifurcation over the femoral head (82.6% vs. 69.8%, *p* < 0.01) [8]. Interestingly, greater experience with ultrasound guidance was not associated with a statistically significant higher rate of successful common femoral artery cannulation. Finally, operators who had performed more than 10 echo-guided procedures had a shorter access time (158 s vs. 268 s *p* < 0.00001) [8].

In a study that included 939 patients who were assigned to either ultrasound-guided access (n = 449) or conventional technique (n = 490), arterial puncture attempts (*p* < 0.001), accidental venipuncture (*p* = 0.02), and total procedure time (*p* = 0.012) were significantly lower in the ultrasound-guided group [9]. Furthermore, the first-pass success rate was significantly higher (*p* < 0.001), and pain levels (*p* < 0.001), hematomas (*p* < 0.001), and arteriovenous fistulas (*p* = 0.011) were significantly lower in the ultrasound-guided group.

The large RCT SURF (Standard versus ultrasound-guided radial and femoral access in coronary angiography and intervention) randomized 1388 patients in a 2 × 2 factorial fashion (radial vs. femoral and standard vs. ultrasound), undergoing coronary angiography and percutaneous coronary intervention [10]. Ultrasound guidance reduced significantly access time (93 s vs. 111 s; *p* = 0.009), number of attempts (1.47 vs. 1.9; *p* < 0.0001), venipuncture (4.1% vs. 9.2%; *p* < 0.0001) and the percentage of cases with difficult access (cases that required five or more attempts) (4.5% vs. 9.2%; *p* = 0.0007) while it improved first-pass success (73% vs. 59.7%; *p* < 0.0001). Nevertheless, the composite primary outcome of ACUITY (Acute Catheterization and Urgent Intervention Triage strategY) is major bleeding, major adverse cardiovascular events (MACE) (death, stroke, myocardial infarction or urgent target lesion revascularization), and vascular complications at 30 days was not reduced in the ultrasound-guided group (*p* = 0.76). It should be noted that ultrasound was used for the guidance of both radial and femoral access [10]. According to an analysis derived from the SURF trial, the minimum number of punctures to gain competency concerning echo-guided femoral access was 15 [11].

The recently published UNIVERSAL (Routine Ultrasound Guidance for Vascular Access for Cardiac Procedures) trial did not show a reduction in bleeding (10% vs. 10.7%, *p* = 0.78 for Bleeding Academic Research Consortium [BARC] 2, 3, or 5 bleeding) with the use of ultrasound in 621 patients undergoing coronary angiography or intervention who were scheduled for femoral access [12] Likewise, there was no statistically significant difference in major vascular complications (6.4% vs. 9.4%, *p* = 0.18) despite the numerical difference. In accordance with the previously mentioned trials, UNIVERSAL showed that ultrasonography improved first-pass success (86.6% vs. 70%, *p* < 0.01), reduced the number of arterial puncture attempts (1.2% vs. 1.4%, *p* < 0.01) and venipuncture (3.1% vs. 11.7%, *p* < 0.01), without adding extra time to the procedure (time to access: 114 s vs. 129 s, *p* = 0.34). A significant limitation of this recent RCT is that very few patients undergoing large bore access were included in this study (2%), with most patients (80%) receiving 6 French introducer size.

A meta-analysis of seven RCTs (not including the recent UNIVERSAL trial), consisting of a total of 3180 patients, reported that compared with the conventional technique, ultrasound-guided cannulation was related to a statistically significant reduction in vascular complications (1.3% vs. 3.0%, *p* = 0.02) and access-site hematoma (1.2% vs. 3.3%; *p* = 0.01), and a non-significant 43% relative risk reduction (absolute risk reduction of 0.7%) in major bleeding events [13]. Investigators of the study noted that they were unable to perform a subgroup analysis in order to examine patients with high-risk clinical characteristics speculating that this subgroup may have even more benefit from the use of ultrasound guidance.

Recently, Iannopolli et al., retrospectively examined access site-related bleeding in 418 patients receiving femoral access (median sheath size 6 F) for multiple combinations of puncture and closure techniques [14]. Access site complications were classified using the BARC criteria. The incidence of bleeding was significantly lower in patients treated with ultrasound-guided access combined with a suture-based closure device.

The UltraCOLOR (ULTrasound-guided TRAnsfemoral puncture in COmplex Large bORe PCΙ, NCT03846752) trial is planning to randomize 542 patients who are going to undergo complex PCI, where 7-Fr or 8-Fr sheaths will be placed, to ultrasound-guided puncture or fluoroscopy-guided puncture with the primary composite endpoint being clinically relevant access site-related bleeding and/or vascular complications requiring intervention [15].

The ongoing REBIRTH (Radial vs. State-Of-The-Art Femoral Access for Bleeding and Access Site Complication Reduction in Cardiac Catheterization, NCT04077762) trial will compare radial access with state-of-the-art femoral access in patients without ST-segment elevation acute myocardial infarction undergoing cardiac catheterization. A second sub-randomization will be performed in the femoral access group with the use of 18 vs. 21-gauge needles. The primary endpoint has been defined as the composite of vascular access complications and bleeding (BARC 2, 3, or 5) up to 30 days post-procedure. REBIRTH aspires to recruit 3266 patients, and if this goal is met, it will become the largest trial on the field.

### 2.2. Ultrasound-Guided Femoral Access for TAVI

The femoral artery is the preferred site of access for TAVI. Given the fact that significantly larger-bore sheaths are used for this procedure in comparison with coronary interventions, ultrasound guidance may offer a higher benefit in terms of the prevention of complications in patients undergoing TAVI. Although randomized data are lacking, multiple observational studies have focused on the usefulness of ultrasound-guided femoral access in terms of complications prevention regarding TAVI procedures.

A retrospective cohort study from Ontario, Canada, included 387 transfemoral TAVI patients, 109 (28%) of whom underwent femoral artery puncture guided by anatomic and angiographic landmarks, whereas 278 (72%) patients had ultrasound-guided puncture [16]. After adjustment for baseline differences, ultrasound-guided access was associated with an odds ratio of 0.42 (95% CI, 0.25–0.70; *p* < 0.01) for the composite endpoint of access-related vascular or bleeding complications and blood transfusion.

A single-center analysis of 154 patients aimed to examine the role of ultrasound-guided femoral puncture for transfemoral TAVI to prevent vascular complications [17]. There was no statistical difference, but a numerical trend for a reduction in major access site-related complications with an ultrasound-guided puncture (4.65% vs. 0.0%; *p* = 0.135).

Another single-center retrospective study of 573 patients compared outcomes and complications between transfemoral TAVI performed with or without ultrasound-guided access [18]. There were no significant differences in in-hospital and one-month mortality between fluoroscopy-guided and ultrasound-guided femoral puncture (1.9% vs. 1.5%, *p* = 0.74 and 3.2% vs. 1.8%, *p* = 0.26). There was no significant difference in minor vascular complications either (12.5% vs. 12.3%, *p* = 1.00). However, major vascular complications were significantly less frequent in the ultrasound-guided group (5.6% vs. 10.2%, *p* = 0.046). Furthermore, after introducing ultrasound-guided puncture, surgical cut-down for transfemoral access was less frequently needed (2.8% vs. 0.3%, *p* = 0.02).

Potluri et al., evaluated femoral access trends and complications from 2012 to 2017 in patients who underwent TAVI [19]. Femoral access was gained under ultrasound guidance in approximately 63% of patients, with ultrasound utilization increasing over time. Patients in the ultrasound-guided group experienced fewer vascular complications than the fluoroscopy roadmap-guided group (7.9% vs. 14.2%, *p* = 0.014). After adjustment for potential confounding risk factors, ultrasound guidance was still associated with a significant reduction in vascular complications (odds ratio 0.51, 95% confidence interval 0.29 to 0.88, *p* = 0.02).

Vincent et al., after performing propensity match scoring, compared 95 patients who had a second-generation valve (Sapien XT [Edwards Lifesciences, Irvine, CA], Corevalve [Medtronic, Dublin, Ireland]) implanted under ultrasound guidance with 95 patients who also had a second-generation valve implanted under fluoroscopy guidance [20] Although the number of patients was relatively small, the authors managed to demonstrate that ultrasound guidance reduced vascular complications (6.3% vs. 16.8%, *p* = 0.023) and life-threatening or major bleeding (6% vs. 22.1%, *p* = 0.004). In a second analysis, a third group of 308 patients who underwent third-generation valve (Evolut-R [Medtronic], Sapien 3 [Edwards Lifesciences]) implantation under ultrasound guidance suffered from a relatively small number of major vascular complications, 3.2% (95% CI, 1.6% to 5.9%) and major bleeding 3.6% (95% CI, 1.8% to 6.3%).

Kotronias et al., used data from the Oxford TAVI (OxTAVI) prospective registry to identify procedural practice predictors of avoidable complications during TAVI [21]. Five-hundred-twenty-nine patients were retrospectively analyzed. The primary endpoint of the study was defined as a hierarchic composite of in-hospital mortality, pericardial effusion/cardiac tamponade, major bleeding, and vascular access complications. The use of ultrasound guidance for vascular access was independently associated with a reduced composite primary endpoint (odds ratio = 0.35, CI: 0.14–0.86, *p* = 0.02) after adjustment for clinical confounders, largely driven by a threefold reduction in vascular access complication (odds ratio = 0.29, CI: 0.15–0.55, *p* < 0.001).

Contrary to the previous studies, Witberg et al., found no benefit from the use of ultrasound guidance in the largest observational study examining femoral access in TAVI patients [22]. Investigators compared fluoroscopy and contralateral angiography versus ultrasound guidance for gaining femoral access in 1171 patients from two different high-volume UK TAVI centers where each center used either the former or the latter technique exclusively. In specific, there was no difference in vascular complications (6.7% vs. 6.8%, *p* = 0.63), bleeding (6.1% vs. 6.4%, *p* = 0.70), or their composite (9.8% vs. 9.8% *p* = 0.76).

A recent metanalysis of the above eight observational studies, which included 3875 patients in total, showed that ultrasound guidance significantly reduced total (Mantel-Haenszel odds ratio [MH-OR], 0.50 [95% CI, 0.35–0.73]), major (MH-OR, 0.51 [95% CI, 0.35–0.74]), and minor (MH-OR, 0.59 [95% CI, 0.38–0.91]) access site vascular complications as well as total access site bleeding complications (MH-OR, 0.59 [95% CI, 0.39–0.90]) [23] The authors concluded that In the absence of randomized studies, their data suggest a potential benefit from ultrasound guidance for femoral access in TAVI.

Due to the large-bore sheaths that are used in TAVI, vascular closure devices are applied by default in such procedures in order to achieve adequate hemostasis. The MANTA (Teleflex, Wayne, Pennsylvania), a collagen-based closure system for large percutaneous arteriotomies, is a commonly used closure device in TAVI procedures [24]. Two studies have compared ultrasound-guided MANTA deployment during TAVI procedures. In the first study, 246 consecutive patients with conventional MANTA deployment and 153 consecutive patients with ultrasound-guided MANTA deployment were evaluated [25]. One-to-one propensity score matching was applied to adequate balance baseline and procedural characteristics and resulted in 135 pairs. Access-site major vascular complications occurred significantly less frequently in patients with ultrasound-guided MANTA (1.5% vs. 7.4%; *p* = 0.030), with a significantly lower incidence of access-site life-threatening or major bleeding complications (1.5% vs. 8.9%; *p* = 0.008). Furthermore, a significantly lower incidence of minor vascular complications (2.2% vs. 8.2%; *p* = 0.028) was observed in the ultrasound-guided MANTA group. In the multivariate analysis, ultrasound-guided MANTA was identified as the only independent predictor of less frequent access-site major vascular complications (OR: 0.29, 95% CI: 0.08 to 0.80). According to the results from the second study, ultrasound-guided MANTA placement reduced MANTA-related vascular complications (6.8% vs. 13%, *p* = 0.001), major or life-threatening bleeding (5.9% vs. 11%, *p* = 0.002), and MANTA failure (3.9% vs. 7.5%, *p* = 0.012) proposing, thus, another application of ultrasonography in TAVI procedures [26].

In summary, although large randomized controlled studies are lacking, the existing evidence supports the use of ultrasound-guided femoral access for percutaneous coronary and aortic valve procedures. Almost all studies having relevant endpoints showed a clear benefit for ultrasound guidance on femoral access technical aspects, such as first-pass success, reduced arterial puncture attempts, and accidental venipuncture. The existing studies are inconsistent regarding clinical outcomes such as vascular complications and bleeding rates. However, when evaluating these data, two important points should be taken into consideration; firstly, that ultrasound-guidance is expected to be more beneficial when large bore sheaths, catheters, and devices are used, and secondly that when the number of events is low (as it happens with major vascular complications and major bleeding), the studies sample sizes need to be large enough to avoid type 2 error.

## 3. The Technique for Ultrasound-Guided Femoral Access

Despite the existing evidence, ultrasound-guided femoral access for coronary and structural percutaneous interventions has not been widely adopted. Surveys of interventional cardiologists demonstrated that only 13% to 27% of operators routinely used ultrasound guidance for femoral access [27,28]. This relatively poor adoption was despite the fact that 88% of the interventional cardiologists answering that ultrasonography was available in their catheterization laboratory. Furthermore, most respondents believed the ultrasound use to be slower than access by palpation alone [27], a belief not proved by the studies previously discussed. Further education in the practical aspects of ultrasound-guided access could improve the technique’s adoption. In this direction, in the following section, we give a detailed description of the technique of femoral arterial access using ultrasound guidance.

The technique of ultrasound-guided femoral puncture is preceded by all the typical steps of femoral arterial access. Firstly, patient co-operation is crucial for a successful outcome. The procedure should be explained in an easily understandable way, and every question to be answered. The patient must feel comfortable to avoid any movements during the procedure. For this reason, if there is any pain (usually back pain) should be promptly treated with analgesic administration, and mild sedation with benzodiazepines is recommended for anxious patients.

Available relevant examinations, such as CT or angiogram of the iliofemoral system, must be reviewed before the procedure. This is a typical step for the TAVI procedure, where a CT aortogram always precedes the operation. Based on the CT, the preferred access site is chosen, and the puncture area is selected. The planned puncture area is at the common femoral artery, above the bifurcation of the superficial and profunda femoral arteries, at the level of the femoral head. When planning the puncture site on the CT, extra care is put into avoiding areas of vessel wall calcification or luminal stenosis.

With the patient on the catheterization table, the presence of femoral artery pulse should be checked before the initiation of the procedure. To confirm the pulse of the right femoral artery, the operator could place his/her left thumb on the right anterior superior iliac spine and the left middle finger on the pubic symphysis indicating the course of the inguinal ligament. The index finger then palpates the femoral artery over the femoral head. Hair in the groin must be shaved, and the area must be sterilized with an antiseptic solution that usually contains iodine (i.e., betadine). A sterile drape is then placed. Both groin areas are scrubbed in case access on the initially selected side is unsuccessful.

**Figure 1:**
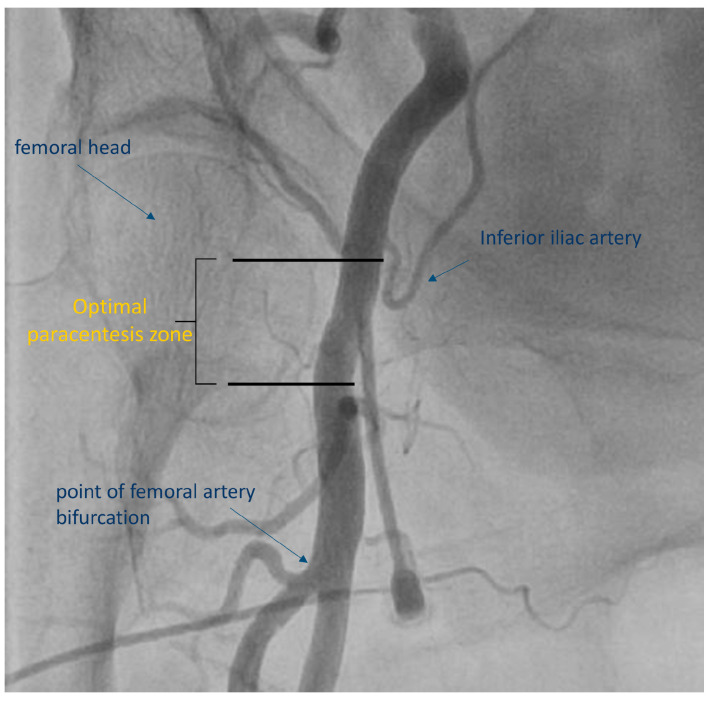
Sheath insertion in the “optimal puncture zone”.

**Figure 2:**
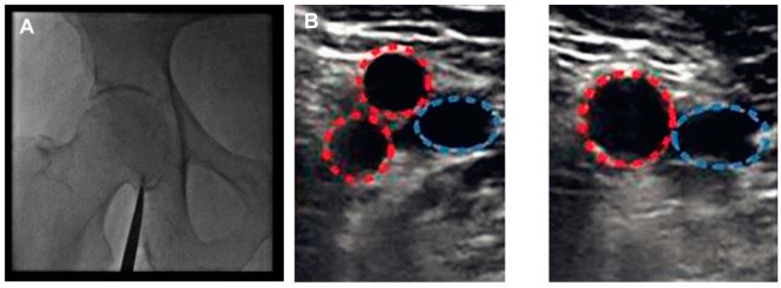
(**A**) Fluoroscopy is used in order to identify the femoral head, and then, the lower edge of the femoral head is marked with a hemostat. (**B**) Ultrasound is used in order to identify the femoral artery. When both superficial and profunda femoral arteries are visible, the probe has been placed under bifurcation (left panel), while when only the common femoral artery is visible, the probe is above femoral bifurcation (right panel), indicating the appropriate puncture site. Common, profunda and superficial femoral arteries are indicated with red color while the femoral vein is indicated with blue color. Reprinted from JACC Cardiovasc Interv. Volume 10, issue 22. Sandoval Y, Burke MN, Lobo AS, et al. Contemporary arterial access in the cardiac catheterization laboratory. Pages 2233–2241, 2017 [31]. Used with permission from Elsevier.

Fluoroscopy of the femoral head can further assist with ultrasound-guided femoral artery puncture [29]. The head of the femoral artery is detected by fluoroscopy, with the center of it being the preferred site of puncture since, after sheath removal, the femoral artery can be compressed against the femoral head, allowing adequate hemostasis. It is estimated that 95–98.5% of the patients have the bifurcation level of the femoral artery at the middle third of the femoral head or lower [8,30]. The ideal zone where femoral artery puncture should take place is over the femoral head and between the common femoral artery bifurcation to the superficial and profunda femoral artery and inferior epigastric artery (Figure 1). A high arterial puncture over the head of the femoral artery (above the origin of the inferior epigastric artery) can result in retroperitoneal hematoma, while a low arterial puncture under the head of the femoral artery (under common femoral artery bifurcation) has been related to pseudoaneurysm formation and arteriovenous fistula [29]. A radiopaque object, such as a hemostat or scissors, is placed at the inferior edge of the femoral head under fluoroscopic guidance in the AP projection (Figure 2, Panel A). A sterile marker can be used in order to draw a line at the level of the inferior edge of the femoral head: attempts to puncture the femoral artery should be performed above this level [31].

**Figure 3:**
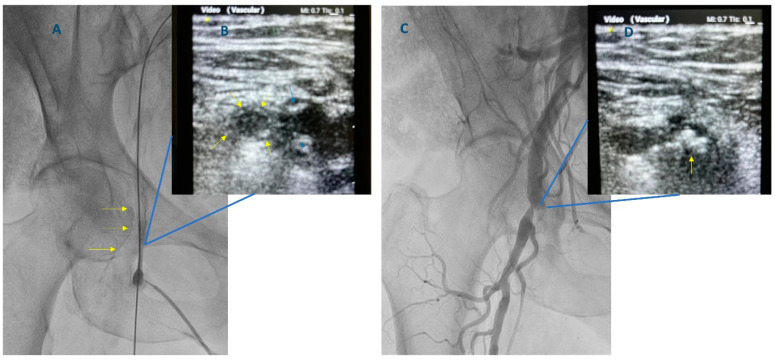
Angiographic and matched ultrasound images of femoral artery wall calcification and superficial femoral artery occlusion in a patient who had Transcatheter Aortic Valve Implantation. (**A**) Fluoroscopy at the level of the right hip shows a significantly calcified right femoral artery wall (arrows). (**B**) Ultrasound confirms the previous finding illustrating circumferential calcification of the profunda (yellow arrows) and calcified plaques in the superficial femoral artery (blue arrows). (**C**) Superficial femoral artery occlusion after the placement of two Perclose Proglide (Abbott Vascular, Redwood City, CA, USA) closure devices. (**D**) Ultrasound illustrates the presence of superficial femoral artery occlusion.

Nevertheless, fluoroscopy cannot predict the level of femoral bifurcation. It is estimated that in one out of three patients, the bifurcation point is over the femoral head. On the other hand, the use of ultrasound can reliably reveal where the common femoral artery is separated into superficial and profunda femoral arteries, reassuring the interventional cardiologist that the point of puncture is above femoral bifurcation [32]. For the ultrasound-guided puncture, a linear vascular ultrasound probe is used (5–12 MHz) [15]. The gel is applied on the tip of the probe, which is inserted in a sterile cover. The operator should ensure that there is no air between the probe and the cover [15]. Sterile ultrasound gel can be applied on the covered probe or the skin. Applying some extra betadine over the patient’s groin can further facilitate ultrasound imaging. The probe is initially placed at the planned puncture area, and the operator starts scanning to identify the common femoral artery and its bifurcation. Depending on the acquired image, the settings for the ultrasound device visualization (i.e., depth and gain) can be optimized. During the whole process, the probe’s long axis should be kept in a perpendicular position to the skin surface. The femoral artery can be visualized in the longitudinal and the short axis view by turning the prone appropriately. The former has the advantage of giving a clear view regarding the point of bifurcation and can also image the femoral head. In the latter view, which is usually the preferred view for a puncture, both the femoral artery and vein are imaged. In this view, the femoral artery and vein appear as an echolucent circle, with the artery found laterally to the vein (Figure 2, Panel B). On contrary to the artery, the vein does not pulsate; however, it can be easily compressed when the probe is pushed against it and could be, in general, larger. Before needle puncture, 10–20 mL of lidocaine is administered under direct ultrasound guidance at the selected site of femoral artery puncture around the artery, avoiding, at the same time, entering the needle into it. The insertion of the needle in the femoral artery should be guided away from heavily calcified or stenosed areas, avoiding, nevertheless, puncturing too high (above the level of the inguinal ligament), a relatively common pitfall for those non-familiar with the ultrasound-guided technique operators. From this perspective, the use of ultrasound to guide the puncture is extremely useful as the presence of vessel wall calcium or vessel stenosis can be clearly imaged and easily identified (Figure 3). The angle that is used for the advancement of the needle into the patient’s groin tissue depends on the distance of the entry point in the patient’s skin from the ultrasound probe (ideally 1 to 3 cm below it). The closer the entry point to the probe, the steeper the angle should be and vice versa [19]. In general, the angle used for needle insertion is 30–45°, but the orientation of the needle can be much steeper, as already explained. The needle entry in the designated location is monitored first by the ‘tenting’ of the vessel in the middle of its ‘dome’ [15]. When the needle enters the artery, brisk pulsatile flow exits its back end. A 0.035-inch wire is then advanced (could be under fluoroscopy guidance). Ultrasound can confirm the appropriate intravascular course of the wire and the fact that the artery puncture was performed above the level of bifurcation. If resistance is felt during advancement, wire further moves forward should be interrupted. The wire is repositioned, under fluoroscopy, until it finds its way to the iliac artery and then to the abdominal aorta. In case of failure, the needle and the wire are removed, and a second puncture attempt should be tried after applying manual pressure to the artery for about 5 min. After successful 0.035-inch wire advancement, the needle is removed, and a (usually 5 to 8 Fr) sheath is inserted over the 0.035-inch wire. A nick with a scalpel can facilitate sheath advancement. The dilator is then removed, and the sheath is aspired and flushed.

A fluoroscopy of the femoral artery is occasionally performed to check the point of insertion and exclude potential complications, such as vessel perforation or dissection. The sheath is connected with the manifold, and femoral artery pressure is recorded. A 20–30 RAO angle for the right and a 20–30 LAO angle for the left femoral artery are used, and the table is moved to have the sheath in the middle of the screen. Then, 5 to 10 mL of contrast or diluted contrast is injected, and the point of sheath insertion, undergrade flow, and the presence of stenosis or complications are appreciated.

**Figure 4:**
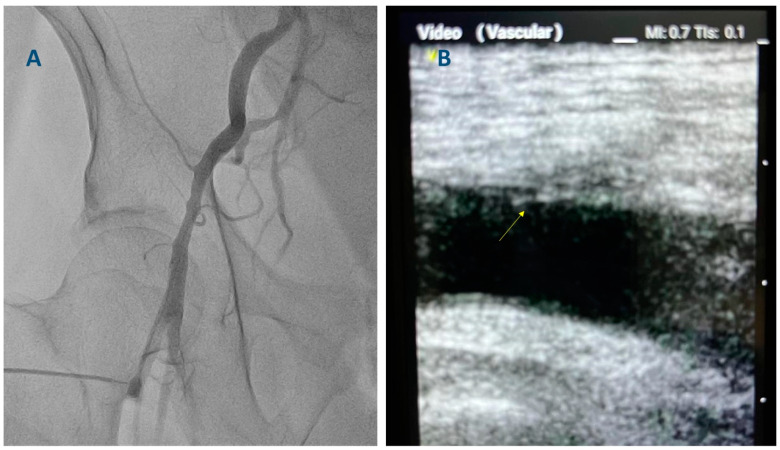
(**A**) Femoral angiography of a patient who will undergo coronary angiography. (**B**) Right femoral imaging with ultrasound in the same patient after coronary angiography completion (longitudinal axis). An Angio-Seal vascular closure device was placed in the right femoral artery (arrow).

After completion of coronary angiography or PCI, the sheath is removed, and hemostasis is achieved with either manual compression or vascular closure devices. Angiographic and ultrasound images of a case where angioseal was used are shown in Figure 4. A detailed description of access closure is beyond the scope of this paper.

## 4. Conclusions

Although radial access has largely replaced femoral access in modern interventional cardiology, the femoral artery remains the preferred or the only site of access for a variety of clinical cases. Ultrasound is inexpensive, lacks complications, and is broadly available. All the previous, in combination with the positive results of the current literature regarding the effectiveness and safety of the technique, argue in favor of routine ultrasound use for gaining femoral access. Ultrasound-guided femoral access is likely to benefit more, patients who undergo operations where large bore sheaths are used, such as complex PCIs and TAVI. Ultrasound-guided femoral access has a steep learning curve, and it should be part of the technical skills to be taught in contemporary interventional cardiology training programs.

## Data Availability

Not applicable.
